# Refractive Changes after Glaucoma Surgery—A Comparison between Trabeculectomy and XEN Microstent Implantation

**DOI:** 10.3390/life12111889

**Published:** 2022-11-15

**Authors:** Caroline Bormann, Catharina Busch, Matus Rehak, Manuela Schmidt, Christian Scharenberg, Focke Ziemssen, Jan Darius Unterlauft

**Affiliations:** 1 Department of Ophthalmology, University of Leipzig, Liebigstrasse 10-14, 04103 Leipzig, Germany; 2 Department of Ophthalmology, University of Giessen, Friedrichstrasse 18, 35392 Giessen, Germany; 3 Augenärzte am Kurpark, Soltauer Straße 6a, 21335 Lüneburg, Germany; 4 Department of Ophthalmology, Inselspital, University of Bern, Freiburgstrasse 4, 3010 Bern, Switzerland

**Keywords:** trabeculectomy, XEN, refraction, visual acuity, surgically induced astigmatism

## Abstract

Best-corrected visual acuity often decreases temporarily or permanently after trabeculectomy (TE). The purpose of this study was to compare visual acuity and refractive changes after TE or XEN microstent implantation (XEN) in primary open-angle glaucoma (POAG) or pseudoexfoliation glaucoma (PEX) cases naïve to prior glaucoma surgery over a 24-month follow-up period. We analyzed 149 consecutive glaucoma patients who received either TE or XEN because of medically uncontrollable POAG or PEX. Intraocular pressure (IOP), IOP-lowering medication use, subjective and objective refraction and best-corrected visual acuity were evaluated. In addition, surgically induced astigmatism (SIA) was calculated and compared using the vector analysis method described by Jaffe and Clayman. A total of 93 eyes (85 POAG; 8 PEX) were treated with TE and 56 eyes (50 POAG; 6 PEX) with XEN. After 24 months, the mean IOP and number of IOP-lowering medications used decreased significantly after TE (*p* < 0.01) and XEN (*p* < 0.01). In the TE group, mean best-corrected visual acuity (BCVA) changed from 0.16 ± 0.26 to 0.23 ± 0.28 logMAR (*p* < 0.01) after 24 months, while mean BCVA did not change significantly in the XEN group (preoperative: 0.40 ± 0.50 logMAR, postoperative: 0.36 ± 0.49 logMAR; *p* = 0.28). SIA was almost the same in both groups at the end of the 24-month follow-up period (0.75 ± 0.60 diopters after TE and 0.81 ± 0.56 diopters after XEN; *p* = 0.57). In addition, there was no significant correlation between SIA and the observed BCVA changes or SIA and IOP reduction 12 or 24 months after TE or XEN. Our results demonstrate that TE and XEN are effective methods for reducing IOP and IOP-lowering medication use. The SIA was nearly similar in both groups. The SIA does not seem responsible for the decreased visual acuity after TE.

## 1. Introduction

After trabeculectomy (TE), we often observe a—partially temporary—reduction in visual acuity, the reasons for which are not yet fully understood. Apart from perioperative pressure peaks, postoperative hypotonic phases or wipe-out syndrome, unexplained visual deterioration remains. But does this also occur after XEN microstent implantation (XEN)?

Glaucoma comprises a heterogeneous group of chronic progressive optic neuropathies. Due to apoptosis of retinal ganglion cells, paracentral (initially relative, later absolute) visual field defects appear and spread to the central visual field in advanced stages of the disease, with concomitant irreversible visual acuity loss [1]. If treated insufficiently, glaucoma may eventually lead to complete blindness. In fact, glaucoma represents one of the major causes of blindness worldwide [2], and the estimated prevalence is 2.9% in Europe and 3.5% worldwide in the age group of 40 years and above [3].

Unalterable risk factors for the occurrence of glaucoma are advanced age, positive family history, higher myopia and the patient’s ethnic origin [4]. Intraocular pressure (IOP) is the most important influenceable risk factor for the development and progression of glaucoma [5,6]. Accordingly, the treatment of glaucoma usually aims to delay its progression by lowering IOP, either through medication and/or surgery. Using the data from one single long-term study (6 years of follow-up) which included 586 eyes with primary open-angle glaucoma (POAG), it was shown that no further increase in visual field defects occurs if an IOP of <18 mmHg could be maintained at all follow-up examinations [7].

TE has been widely accepted as the gold standard in glaucoma surgery for more than 40 years [8]. Additionally, minimally invasive glaucoma surgery (MIGS) evolved in order to minimize surgical trauma compared to TE and overcome the different possible complications which were seen after TE [9]. In addition, the advantages of MIGS are the shorter operating time, lower postoperative discomfort and shorter convalescence times. One representative of MIGS is the XEN45 Gel Stent (XEN, Allergan, Irvine, CA, USA), which consists of a flexible 6 mm long tube made from pig gelatin. XEN connects the anterior chamber with the subconjunctiva or, even better, with the subtenon space and allows aqueous humor to leave the anterior chamber if the pressure gradient exceeds 5 mmHg [10].

There is often a (temporary) visual loss or refractive change during the first year after TE [11], which may be caused by the development of a cataract, necessary re-operations, postoperative choroidal detachment or maculopathy [12]. MIGS aspires to effectively reduce IOP while being less traumatic at the same time. There have been multiple studies showing the efficacy of MIGS in terms of the reliable reduction in IOP and/or in the use of necessary IOP-lowering eye drops [13,14,15]. The aim of this study was to evaluate the 24-month outcomes of visual acuity, refractive changes and surgically induced astigmatism (SIA) in 149 glaucoma patients who received either TE or XEN.

## 2. Materials and Methods

This study was designed as a monocentric, retrospective, comparative analysis of 149 glaucoma patients. All POAG or PEX patients were treated by the same ophthalmic surgeon with TE or XEN (XEN, Allergan, Irvine, CA, USA) between January 2017 and March 2019 at the University Eye Hospital in Leipzig, Germany. The study was approved by the local Ethics Committee (209/18-ek) and the Declaration of Helsinki was respected at all times.

Inclusion criteria were pseudophakia, no prior incisional glaucoma surgery and verified presence of POAG or PEX glaucoma with typical optic disc changes (pathologically increased cup-to-disc ratio of >0.4 depending on the area of the optic disc and significant thinning of the papillary neuroretinal nerve fiber layer). Additional requirements were an IOP repeatedly measured above individual target pressure by applanation tonometry, despite the maximum tolerable medication, and increasing scotomas or an increase in mean deviation (2 dB/year) in the static-automated visual field examination (during the last 12 months before surgery). Exclusion criteria were patient age <35 years, every glaucoma entity other than POAG or PEX glaucoma and the necessity for combined glaucoma and cataract surgery. TE was performed in eyes with an IOP >40 mmHg or cases of a visual acuity >0.2 logMAR in the contralateral eye due to the so far reported more favorable safety profile of TE compared to XEN. All other eyes received a XEN microstent implantation.

The decision for TE or XEN was made during an ambulant examination and in consideration of the individual IOP-lowering medication. The examination included an extensive ophthalmologic status assessment, including objectively (ARK-1s autorefractor, NIDEK, Aichi, Japan) and subjectively adjusted refraction, best-corrected visual acuity (BCVA) using Snellen charts (converted into logMAR for statistical analysis) and Goldmann applanation tonometry, as well as an inspection of the anterior and posterior chamber and an examination of the chamber angle using a Sussmann gonioscope (Ocular Instruments INC, Bellevue, WA, USA). In addition, visual field assessment was performed using standard automated perimetry (Twinfield 2, Oculus Optikgeräte GmbH, Wetzlar, Germany; 24-2 test strategy, 55 test locations). Additional patient symptoms and ophthalmic and general medical histories were collected, but no comorbidities were taken into account for the data analysis performed. All examinations were performed again on the day of admission to the hospital.

Four weeks before the planned operation date, all IOP-lowering eye drops were stopped and a therapy using steroid eye drops (prednisolone acetate 1%, BID for 4 weeks) and systemic carbonic anhydrase inhibitors (acetazolamide 250 mg, BID for 4 weeks) was started, which was used until the operation date.

### Surgical Procedures

The XEN microstent was implanted as commonly performed into the upper nasal quadrant using two 1.1 mm paracenteses. One paracentesis was located opposite to the planned implantation site (temporal-inferior) and the other paracentesis was located nasally to stabilize the eye during implantation of the XEN. TE was performed according to the fornix-based technique with a 4 × 4 mm scleral flap using 2–4 10/0 non-absorbable sutures (Ethilon 10/0, Ethicon, Johnson & Johnson Medical GmbH, Norderstedt, SH, Germany). To readapt the conjunctiva to the limbus, we usually used 4 absorbable single button sutures (Vicryl 10/0, Ethicon, Johnson & Johnson Medical GmbH, Norderstedt, SH, Germany).

Postoperative therapy after TE or XEN included antibiotic (gentamicin; QID for 1 week), cycloplegic (atropine 1%; BID for 1 week after XEN and depending on clinical assessment after TE) and steroid eye drops (prednisolone acetate 1%; QID for 4 weeks, titrated thereafter depending on clinical assessment).

The following data were collected before surgery and during the postoperative examination 3, 6, 12 and 24 months after surgery: IOP, number of applied IOP-lowering drugs, BCVA, mean deviation (MD) of standard automated perimetry (only 6, 12 and 24 months after surgery) and objective and subjective refraction. Additionally, the anterior and posterior segments of the eyes were examined using a slit lamp. Finally, surgically induced astigmatism (SIA) was calculated using the vector method described by Jaffe and Clayman.

The programs Excel (Version 2007, Microsoft; Redmond, DC, USA) and SPSS (IBM Version 22.0; Chicago, Illinois, IL, USA) were used for data acquisition and statistical analysis. For patient age, IOP, IOP-lowering medication, BCVA, MD, sphere and SIA, the mean and standard deviation were calculated. We used the non-parametric Wilcoxon test and the Mann–Whitney U test for statistically significant differences (*p* ≤ 0.05) in the postoperative course. In addition, visual acuity, SIA and IOP reduction were tested for possible correlation by using the Pearson’s test.

## 3. Results

In the present study, 93 eyes of 93 patients were treated with TE and 56 eyes of 56 patients with XEN implantation. For all 149 eyes, a 24-month follow-up could be obtained. Preoperative patient characteristics were comparable between both subgroups. Only the differences in visual acuity and astigmatism were statistically significant before surgery. Mean visual acuity was lower and preoperative astigmatism higher in the XEN-group (Table 1).

### 3.1. IOP, IOP-Lowering Medication

**Figure 1:**
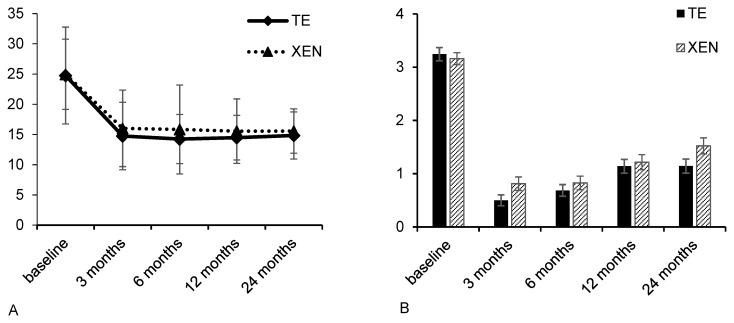
Development of IOP (**A**) and number of applied IOP-lowering medications (**B**) during the 24 months after TE or XEN.

The preoperative IOP was comparable in both subgroups (TE: 24.8 ± 8.0 mmHg; XEN: 25.0 ± 5.8 mmHg; intergroup comparison *p* = 0.49). The IOP decreased significantly compared to baseline in both groups during the 24-month postoperative period and did not differ significantly between the two groups for all follow-up examinations (Figure 1 and Table 2). Twelve and twenty-four months after TE, the mean IOPs were 14.5 ± 3.7 mmHg (*p* < 0.01) and 14.8 ± 3.9 mmHg (*p* < 0.01), respectively. In the XEN group, the mean IOP dropped to 15.6 ± 5.3 mmHg (*p* < 0.01) and 15.6 ± 3.7 mmHg (*p* < 0.01) over the same period. Preoperatively, the mean number of applied IOP-lowering medications was 3.3 ± 1.3 in the TE group and 3.2 ± 1.1 in the XEN group, with no significant difference between the two subgroups (*p* = 0.89). After 12 and 24 months, they were reduced significantly to 1.1 ± 1.3 (*p* < 0.01) and 1.1 ± 1.3 (*p* < 0.01) in the TE group and to 1.2 ± 1.4 (*p* < 0.01) and 1.5 ± 1.5 (*p* < 0.01) in the XEN group (Table 2 and Figure 1). There was no statistically significant difference between the two groups during follow-up examinations (Table 2).

### 3.2. Visual Acuity, Visual Field

The mean BCVA was 0.16 ± 0.26 logMAR in the TE group and 0.40 ± 0.50 logMAR in the XEN group at baseline examination, thus showing a statistically significant difference between the two subgroups (*p* < 0.01), so no further intergroup comparisons were performed. To facilitate comparison of BCVA between the TE and XEN groups, results were stratified into three groups depending on visual acuity at baseline (group 1: BCVA < 0.4 logMAR; group 2: BCVA 0.4–1.0 logMAR; group 3: BCVA > 1.0 logMAR). In comparing the BCVA results between the TE and XEN groups, no statistically significant difference was detectable at baseline nor at any follow-up visit (Table 3).

In the TE group only, the intragroup comparison demonstrated a significant decrease in the mean visual acuity to 0.21 ± 0.24 logMAR (*p* < 0.01) and 0.23 ± 0.28 logMAR (*p* < 0.01) at 12 and 24 months postoperatively, respectively. In contrast, there was no statistically significant difference compared to baseline in the XEN group (12 months: 0.41 ± 0.48 logMAR; *p* = 0.37 and 24 months: 0.36 ± 0.49 logMAR; *p* = 0.28; Table 2).

Visual field examinations showed a mean defect of 8.16 ± 4.87 dB in the TE group and of 10.36 ± 3.80 dB in the XEN group, with a significant difference between the two groups at baseline (*p* = 0.02). However, the mean MD did not change significantly over the 24-month follow-up in both groups (TE group 12 months: *p* = 0.71; 24 months: *p* = 0.69; XEN group 12 months: *p* = 0.10; 24 months: *p* = 0.11; Table 2).

### 3.3. Sphere, SIA

**Figure 2:**
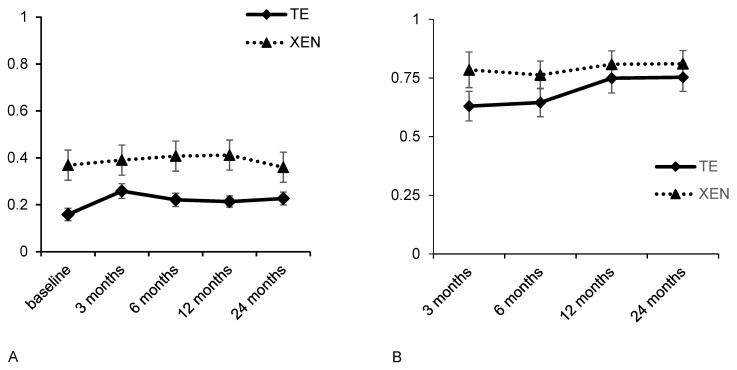
Development of best-corrected visual acuity (**A**) and SIA (**B**) during the 24 months after TE or XE.

In the TE group, the mean sphere was −0.38 ± 2.73 dpt at baseline, and −0.44 ± 3.06 dpt and −0.31 ± 2.89 dpt 12 and 24 months after surgery, respectively. In comparison, the mean sphere was −0.46 ± 1.97 dpt at baseline, and −0.23 ± 1.36 dpt and −0.29 ± 1.45 dpt at 12 and 24 months after XEN, respectively. In both groups, changes in mean sphere did not reveal statistically significant differences at any of the follow-up examinations (Table 4). The SIA was calculated using the vector analysis proposed by Jaffe and Clayman (for details, see Table 4 and Figure 2). In both groups, significant SIA could be observed 3 months postoperatively (TE: 0.63 ± 0.65; XEN: 0.79 ± 0.76; TE and XEN *p* < 0.01). However, 6, 12 and 24 months after glaucoma surgery there were no further significant changes in the existing SIA. At the 24-month follow-up examination, the SIA was 0.75 ± 0.60 dpt after TE and 0.81 ± 0.56 dpt after XEN. Further analysis showed no statistically significant differences between the TE and XEN groups 6, 12 and 24 months after surgery (6 months postoperatively: *p* = 0.24; 12 months postoperatively: *p* = 0.46; 24 months postoperatively: *p* = 0.57).

In addition, the observed SIA and BCVA changes were tested for a possible correlation, without any significant results 12 or 24 months after TE or XEN (TE group 12 months: r = −0.05, *p* = 0.71; 24 months: r = −0, 06, *p* = 0.61 and XEN group 12 months: r = −0.23, *p* = 0.12; 24 months: r = −0.11, *p* = 0.48). Furthermore, a possible correlation between the resulting SIA and the operative IOP reduction was examined, though no significant correlations were found in the TE group nor in the XEN group for the results obtained 12 or 24 months postoperatively (TE group 12 months: r = −0.24, *p* = 0.85; 24 months: r = −0.02, *p* = 0.88; XEN group: 12 months: r = −0.17, *p* = 0.25; 24 months: r = −0.33, *p* = 0.30).

## 4. Discussion

Our study demonstrates an effective reduction in IOP and in the number of applied IOP-lowering drugs 2 years after TE or XEN in POAG or PEX eyes naïve to prior glaucoma surgery. Additionally, measurable postoperative refraction changes and similarly pronounced SIA were observed after both surgical procedures. In fact, significant reductions in IOP and in the number of necessary IOP-lowering medications after TE or XEN implantation have already been shown in a large number of different mono- and multi-center trials with different follow-up lengths [16,17,18,19]. In addition, the effectiveness of both methods was also compared in one separate study of our own [20].

The focus of this study was to evaluate and compare the emerging SIA after XEN or TE. Here, we observed a SIA of 0.75 ± 0.6 dpt after TE and 0.81 ± 0.56 dpt after XEN, each measured 24 months postoperatively. This is in line with results of previously published studies, which, in addition to changes in refraction, described a mean SIA of around 1 diopter 6 months after TE [21,22,23,24]. Even though the induction of postoperative astigmatism has not been explicitly ruled out for the new emerging MIGS procedures, it could be assumed that the minor surgical trauma, and especially the absence of transscleral sutures, could result in a lower SIA after XEN than TE. Comparative studies between TE and non-penetrating glaucoma surgery (microstent implantation or shunt surgery) showed the resulting SIA was more pronounced after TE [25,26]. However, in our study the SIA was nearly similar in both groups. Furthermore, in the literature, a significantly higher SIA was described in the first days after TE, which subsequently decreased during the first 12 months after surgery [27,28]. Our data also show measurable SIA even in the early postoperative phase (3 months), which did not change during the later examination dates at 6, 12 or 24 months after surgery.

Various explanatory approaches for the observed SIA after glaucoma surgeries have been discussed before. First, a possible correlation between postoperative IOP reduction and SIA was described [25,29]. Delbeke et al. also observed this correlation 1 month after TE + MMC, but it was no longer detectable 6 months after surgery [30]. Reasons could be an IOP-, bleb- or suture-associated deformation of the cornea in the early postoperative phase. Based on our results, no significant correlation between IOP and SIA could be found 12 and 24 months postoperatively. This is in line with data from El-Saied et al., who also found no significant correlation between postoperative astigmatic change and IOP reduction after TE and deep sclerectomy [23]. Other possible reasons for SIA after TE are extensive scleral cautery or corneal steepening of the vertical corneal radius due to a destabilization around the scleral flap [24]. Our results are not in line with this assumption because of the nearly identical SIA after TE as well as XEN. Third, wound-healing processes influenced by MMC are possible. One study showed that SIA was less pronounced 12 months after TE + MMC compared to TE without MMC [31]. Fourth, postoperative ptosis and the resulting conjunctival bleb were considered as an explanation for the nearly similar SIA after TE and XEN in our study. Tear film and SIA could be affected by the conjunctival bleb after both surgical procedures. In the case of XEN, the insertion of the implant and the anatomical changes induced in the sclerocorneal junction or the postoperative inflammation could have also influenced SIA. In the future, pre- and postoperative topographical analysis of the cornea might be helpful to assess the possible influence of the bleb on the resulting SIA after TE and XEN. In addition, other factors influencing SIA after TE and XEN should be examined.

Visual acuity significantly decreased 3 months postoperatively solely in the TE group and not in the XEN group. Subsequently, visual acuity remained stable during follow-up. The loss of visual acuity could be due to the intensive postoperative application of local steroids and atropine or necessary suturolysis after TE. The natural course of postoperative cataract development, which may be enhanced by surgical trauma and postoperative inflammation, is also believed to be a common cause for visual loss after TE [12]. Therefore, only already pseudophakic eyes were included in the current study group and no cases of combined glaucoma and cataract surgery were allowed. Furthermore, the sutures at the superior limbus in TE could possibly cause an irregular astigmatism, which cannot be demonstrated by the calculated SIA. A randomized prospective study in Mainz observed faster visual rehabilitation through suture removal of the conjunctival sutures after TE (fornix-based flap). However, no significant effect on the resulting SIA was shown [28]. It could be considered that removal of the sutures reduced the induced irregular astigmatism, allowing for faster visual rehabilitation. It seems reasonable that the reduced visual acuity after TE in our study may predominantly be due to an induced irregular astigmatism.

Accordingly, best-corrected visual acuity in our study remained stable after XEN. An unaltered visual acuity had also been demonstrated by Dehghanian 3 months after shunt surgery [26]. The stable visual acuity after XEN compared to TE could be seen as a criterion for XEN being less traumatic than TE.

To increase group comparability, only eyes with POAG or PEX glaucoma were analyzed. In addition, the large number of eyes (*n* = 93 in the TE group, *n* = 56 in the XEN group) and the long follow-up period of 2 years should be emphasized. Furthermore, we were able to compare two different surgical procedures and show that even after XEN using two 1.1 mm paracenteses, there was a measurable change in refraction and a mean SIA of 0.81 ± 0.56 dpt present 24 months after surgery. Major limitations of our study are its retrospective, monocentric, non-blinded and non-randomized design.

In the future, further studies examining the effect of MMC on the postoperative change in refraction or performing topographical analysis of the cornea in the postoperative course to investigate the suspected induced irregular astigmatism after TE will certainly be helpful, as will studies including combined cases of MIGS and cataract surgery.

## 5. Conclusions

After TE and XEN, we found significant and comparable SIA 3 months after surgery, which remained stable during the further follow-up period of 24 months. In any case, a significant and seemingly permanent refractive change occurred after both procedures, which patients should be informed about before surgery.

## Figures and Tables

**Table 1:** Baseline patient characteristics of all eyes treated with TE or XEN.

	TE	XEN	Mann–Whitney U Test *p*=
**Age [years]**	70.4 ± 8.8	70.3 ± 10.7	0.58
**n**	93	56	
**Gender**	♀: 52; ♂: 41	♀: 30; ♂: 26	0.71
**Laterality**	right: 48; left: 45	right: 26; left: 30	0.61
**Glaucoma**	POAG: 85; PEX: 8	POAG: 50; PEX: 6	0.52
**IOP (mmHg)**	24.8 ± 8.0	25.0 ± 5.8	0.49
IOP Female (mmHg)	22.7 ± 11.9	24.8 ± 13.5	0.11
IOP Male (mmHg)	28.0 ± 14.7	25.1 ± 13.3	0.21
**Best-corrected visual acuity [logMAR]**	0.16 ± 0.26	0.40 ± 0.50	**<0.01**
**Sphere [dpt]**	−0.38 ± 2.73	−0.46 ± 1.97	0.54
**Astigmatism [dpt]**	−0.91 ± 0.77	−1.33 ± 0.96	**<0.01**

TE: trabeculectomy, XEN: XEN microstent implantation, POWG: primary open-angle glaucoma, PEX: pseudoexfoliative glaucoma, IOP: intraocular pressure, dpt: diopters.

**Table 2:** Baseline and follow-up results for mean IOP, number of applied IOP-lowering medications, best-corrected visual acuity and MD during the 24 months after TE or XEN.

		TE	Comparison to Baseline (Wilcoxon Test) *p*=	XEN	Comparison to Baseline (Wilcoxon Test) *p*=	Intergroup Comparison (Mann–Whitney U Test) *p*=
**IOP [mmHg]**	**baseline**	24.8 ± 8.0	n.a.	25.0 ± 5.8	n.a.	0.49
	**3 months**	14.7 ± 5.6	**<0.01**	16.0 ± 6.3	**<0.01**	0.16
	**6 months**	14.2 ± 4.1	**<0.01**	15.8 ± 7.4	**<0.01**	0.31
	**12 months**	14.5 ± 3.7	**<0.01**	15.6 ± 5.3	**<0.01**	0.28
	**24 months**	14.8 ± 3.9	**<0.01**	15.6 ± 3.7	**<0.01**	0.32
**Medication [n]**	**baseline**	3.3 ± 1.3	n.a.	3.2 ± 1.1	n.a.	0.89
	**3 months**	0.5 ± 1.0	**<0.01**	0.8 ± 1.3	**<0.01**	0.07
	**6 months**	0.7 ± 1.1	**<0.01**	0.8 ± 1.3	**<0.01**	0.55
	**12 months**	1.1 ± 1.3	**<0.01**	1.2 ± 1.4	**<0.01**	0.77
	**24 months**	1.1 ± 1.3	**<0.01**	1.5 ± 1.5	**<0.01**	0.15
**Best-corrected visual acuity [logMAR]**	**baseline**	0.16 ± 0.26	n.a.	0.40 ± 0.50	n.a.	**<0.01**
	**3 months**	0.26 ± 0.31	**<0.01**	0.39 ± 0.48	0.19	n.a.
	**6 months**	0.22 ± 0.28	**<0.01**	0.41 ± 0.50	0.36	n.a.
	**12 months**	0.21 ± 0.24	**<0.01**	0.41 ± 0.48	0.37	n.a.
	**24 months**	0.23 ± 0.28	**<0.01**	0.36 ± 0.49	0.28	n.a.
**MD [dB]**	**baseline**	8.16 ± 4.87	n.a.	10.36 ± 3.80	n.a.	**0.02**
	**6 month**	7.84 ± 4.91	0.51	11.48 ± 3.85	0.09	n.a.
	**12 month**	8.07 ± 5.30	0.71	11.71 ± 3.60	0.10	n.a.
	**24 month**	7.68 ± 5.03	0.69	11.65 ± 3.58	0.11	n.a.

TE: trabeculectomy, XEN: XEN microstent implantation, IOP: intraocular pressure, n.a. = not applicable, MD: mean deviation, dB: decibel.

**Table 3:** Baseline and follow-up results for best-corrected visual acuity stratified into three groups.

		TE	XEN	Intergroup Comparison (Mann–Whitney U Test) *p*=
**Visual acuity < 0.4** **logMAR**	**baseline**	0.09 ± 0.13	0.14 ± 0.14	0.18
	**3 months**	0.21 ± 0.26	0.20 ± 0.17	0.67
	**6 months**	0.18 ± 0.26	0.18 ± 0.18	0.63
	**12 months**	0.17 ± 0.21	0.21 ± 0.20	0.23
	**24 months**	0.17 ± 0.23	0.18 ± 0.15	0.43
**Visual acuity 0.4–1.0** **logMAR**	**baseline**	0.61 ± 0.13	0.54 ± 0.07	0.32
	**3 months**	0.69 ± 0.17	0.56 ± 0.24	0.21
	**6 months**	0.70 ± 0.16	0.66 ± 0.22	0.54
	**12 months**	0.65 ± 0.20	0.65 ± 0.17	0.89
	**24 months**	0.73 ± 0.30	0.82 ± 0.67	0.54
**Visual acuity > l.0** **logMAR**	**baseline**	1.9 ± 0.51	1.63 ± 0.43	0.86
	**3 months**	1.5 ± 0.55	1.62 ± 0.61	0.67
	**6 months**	1.7 ± 0.45	1.58 ± 0.57	0.58
	**12 months**	1.32 ± 0.60	1.54 ± 0.64	0.62
	**24 months**	1.51 ± 0.63	1.40 ± 0.69	0.73

TE: trabeculectomy, XEN: XEN microstent implantation.

**Table 4:** Baseline and follow-up results for mean sphere and SIA during the 24 months after TE or XEN.

		TE	Comparison to Baseline (Wilcoxon Test) *p*=	XEN	Comparison to Baseline (Wilcoxon Test) *p*=	Intergroup Comparison (Mann–Whitney U Test) *p*=
**Sphere [dpt]**	**baseline**	−0.38 ± 2.73	n.a.	−0.46 ± 1.97	n.a.	0.54
	**3 months**	−0.47 ± 2.68	0.27	−0.32 ± 1.29	0.95	0.48
	**6 months**	−0.49 ± 2.79	0.69	−0.18 ± 1.25	0.19	0.34
	**12 months**	−0.44 ± 3.06	0.29	−0.23 ± 1.36	0.59	0.27
	**24 months**	−0.31 ± 2.89	0.15	−0.29 ± 1.45	0.21	0.38
**SIA [dpt]**	**3 months**	0.63 ± 0.65	**<0.01**	0.79 ± 0.76	**<0.01**	0.40
	**6 months**	0.65 ± 0.61	0.74	0.76 ± 0.59	0.95	0.24
	**12 months**	0.75 ± 0.63	0.19	0.81 ± 0.57	0.75	0.46
	**24 months**	0.75 ± 0.60	0.15	0.81 ± 0.56	0.66	0.57

SIA: surgically induced astigmatism, TE: trabeculectomy, XEN: XEN microstent implantation, n.a. = not applicable, dpt: diopters.

## Data Availability

Datasets generated during the current study are available from the corresponding author upon reasonable request.
